# The relationship of cortical folding and brain arteriovenous malformations

**DOI:** 10.1186/s40809-016-0024-3

**Published:** 2016-11-22

**Authors:** Manish N. Shah, Sarah E. Smith, Donna L. Dierker, Joseph P. Herbert, Timothy S. Coalson, Brent S. Bruck, Gregory J. Zipfel, David C. Van Essen, Ralph G. Dacey

**Affiliations:** 1 Departments of Pediatric Surgery and Neurosurgery, McGovern Medical School at UT Health and UT MD Anderson Cancer Center, Pediatric Neurosurgery, 6431 Fannin St., MSB 5.144, Houston, TX 77030, USA.; 2 Department of Neuroscience, Washington University, 660 S. Euclid Ave, St. Louis, MO 63110, USA.; 3 Division of Neurosurgery, University of Missouri-Columbia, One Hospital Drive, 314 McHaney Hall, Columbia, MO 65212, USA.; 4 Department of Neurological Surgery, Washington University, 660 S. Euclid Ave, St. Louis, MO 63110, USA.

**Keywords:** AVM, MRI, Cortical folding, *de novo*, Congenital, Brain development

## Abstract

**Background:**

The pathogenesis of human intracranial arteriovenous malformations (AVMs) is not well understood; this study aims to quantitatively assess cortical folding in patients with these lesions.

**Methods:**

Seven adult participants, 4 male and 3 female, with unruptured, surgically unresectable intracranial AVMs were prospectively enrolled in the study, with a mean age of 42.1 years and Spetzler-Martin grade range of II–IV. High-resolution brain MRI T1 and T2 sequences were obtained. After standard preprocessing, segmentation and registration techniques, three measures of cortical folding, the depth difference index (DDI), coordinate distance index (CDI) and gyrification index (GI)), were calculated for the affected and unaffected hemispheres of each subject as well as a healthy control subject set.

**Results:**

Of the three metrics, CDI, DDI and GI, used for cortical folding assessment, none demonstrated significant differences between the participants and previously studied healthy adults. There was a significant negative correlation between the DDI ratio between affected and unaffected hemispheres and AVM volume (correlation coefficient *r* = −0.74, *p* = 0.04).

**Conclusion:**

This study is the first to quantitatively assess human brain cortical folding in the presence of intracranial AVMs and no significant differences between AVM-affected versus unaffected hemispheres were found in a small dataset. We suggest longitudinal, larger human MRI-based cortical folding studies to assess whether AVMs are congenital lesions of vascular development or *de novo*, dynamic lesions.

## Background

Intracranial arteriovenous malformations (AVMs) are the direct communication of arteries to abnormally tortuous and dilated veins without an interposing capillary bed, often described as a tangle or “bag of worms” [1]. The mechanism of AVM formation is not well understood. Unlike their pulmonary or abdominal counterparts, brain AVMs have long been thought to be congenital malformations situated in often eloquent, functional brain parenchyma. Due to this direct, high-pressure, high-flow connection, patients are subject to hemorrhage, seizures and strokes [2]. If AVMs are truly congenital lesions of the intracranial vascular system, there is a poorly understood interaction between the processes of AVM formation and cortical folding. Although the large vessels in the intracranial vascular system are mostly formed and have perforated the cortex by weeks 8–10 in utero, the brain begins its intricate folding process at weeks 24–34 (reviewed in [3–5]). To date, no hypothesis or human study explains what happens in the 16 week interval between intracranial vasculature maturation and cortical folding initiation [6]. There are no experimental animal AVM models within the brain parenchyma that expose either the mechanism of formation or the effect on cortical folding [7–10].

Cortical folding is a complex phenomenon that remains poorly understood. Our center has discovered cortical folding abnormalities in other diseases of the neurological system such as Williams’ syndrome [11] and autism spectrum disorder [12]. Beginning at approximately 26 weeks gestation, cortical folding is thought to rely on a variety of factors including, but not limited to, gene expression, cortical growth, and tension from white matter fibers [13]. In addition, adequate oxygenation is necessary for proper neurodevelopment and cortical folding. Maintenance of tissue perfusion and adequate oxygenation relies heavily on changes at the microvascular level in response to various physiologic cues. The neuronal vasculature in particular is exquisitely sensitive to such signals, which include CO_2_ and O_2_ levels, mechanical distention and compression, and changes in local neuronal activity [14]. Dysregulation of the microvascular response to these factors results in an area of hypoperfusion. Brain tissue in an affected area is most at-risk for acute infarct or hypoperfusion when surrounding vasculature dilates in order to meet metabolic demand. Cortical steal syndrome occurs when increased resistance in the brain parenchyma relative to the surrounding normal vasculature causes a paradoxical drop in local perfusion pressure.

The extent to which AVMs induce this degree of local hypoxia is debated [2]. Cerebral hypoperfusion has been demonstrated in some patients [15], but not in others [16]. It appears that the main mechanism for maintenance of perfusion in brain surrounding AVMs in the latter group is neo-capillary formation [17]. This phenomenon is presumably the result of some initial hypoxic state that induces local vasculogenesis. Hypoxia and cerebral ischemia can cause cortical thinning due to selective neuronal loss [18–20]. Fierstra et al. demonstrated that cortical thinning is also seen in patients with cortical steal syndrome [21]. They proposed that repeated bouts of transient hypoperfusion associated with steal physiology, while insufficient to cause acute infarction, leads to selective neuronal loss over time.

It is likely that AVMs cause local tissue hypoxia at least initially and, as vasculogenesis precedes cortical folding by several months [6], AVMs could impact cortical development. The human brain has an overwhelming tendency to form complex cortical folds that are orderly in some respects but show a high degree of individual variability in most regions. If AVMs are true congenital lesions formed prior to cortical involution, their mechanical traction on adjacent brain parenchyma should substantially alter the process of cortical folding. This study aims to assess human cortical folding patterns with AVMs using advanced measurements of cortical shape and high-resolution MRI.

## Methods

### Participants

With institutional review board approval and informed consent, participants were prospectively enrolled in the study. A total of 7 otherwise healthy patients with surgically unresectable AVMs, incidentally found or presenting with seizures but without hemorrhage were enrolled. In addition, one healthy adult control subject was also enrolled, but was not used for the analysis. Some patients had recent stereotactic, focal irradiation to the AVM bed for treatment with Gamma Knife Radiosurgery, but there was no appreciable radiation effect on MRI.

The subject demographics and clinical characteristics are summarized in Table 1. The 7 subjects had a mean age of 42.1 years with 3 females and 4 males. The Spetzler-Martin grades ranges from II–IV. Five subjects initially presented incidentally or with headaches, one with seizures and one with facial numbness. Four of seven were treated with gamma knife stereotactic radiation, one had a hemorrhage and subsequent surgical resection and two had refused radiation treatment at last follow-up. Of the 5 treated patients, 3 had a positive response or elimination of the AVM, one subject had radiographic AVM growth and one subject expired after AVM rupture and hemorrhage. Overall, the seven subjects had an average clinical follow-up time of 17.8 months from last treatment.

**Fig. 1:**
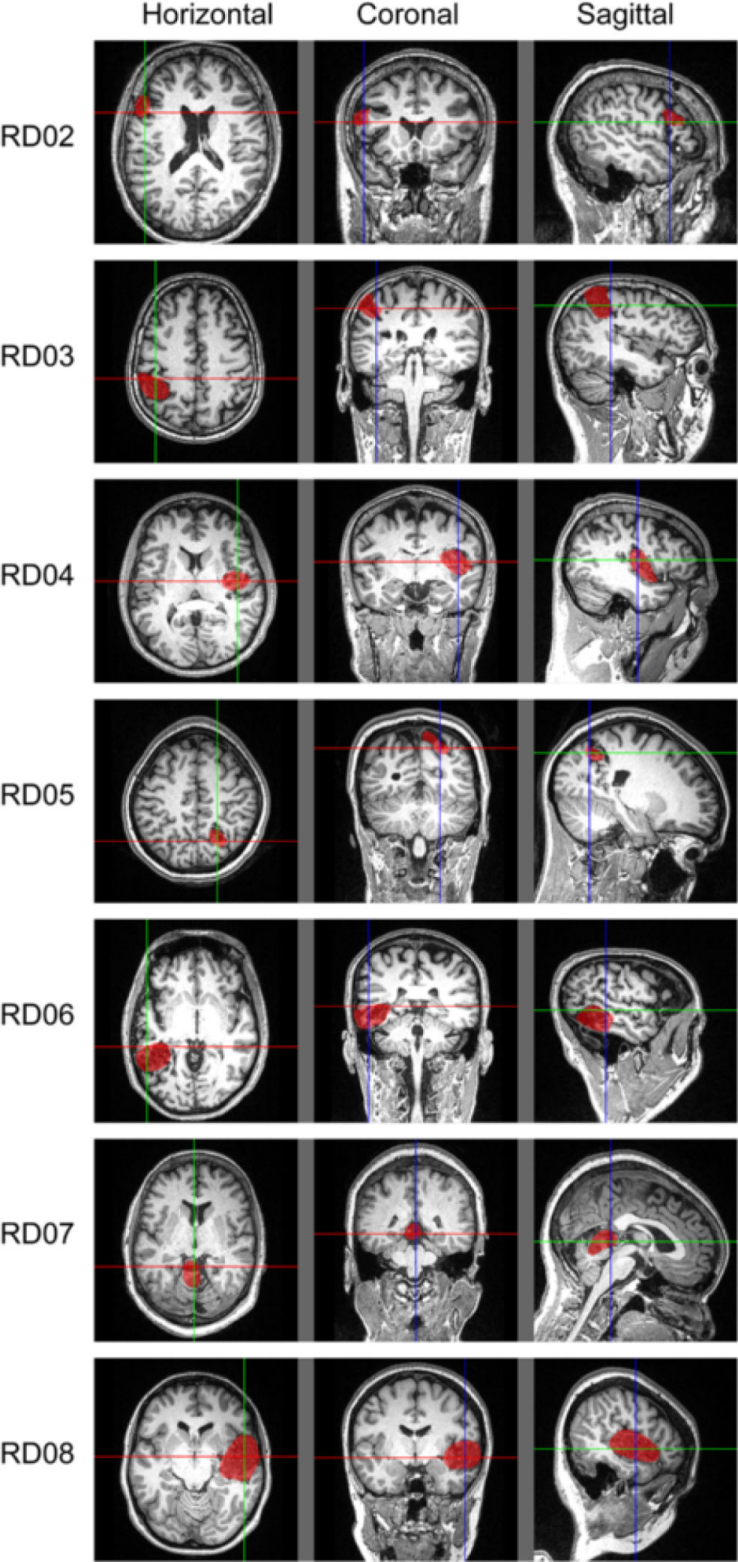
AVM Location on T1-weighted Horizontal, Coronal and Sagittal MRIs in anatomical space. Image right is anatomical right. For each subject, the pre-surgical T1w MRIs are displayed. Colored lines orient location of each MRI view: green runs front-back, red runs left-right and blue runs up-down. AVMs were traced for distance-from-AVM analysis and the inside of the AVM is shaded red for visualization

The AVMs from the seven subjects varied greatly in terms of volume and location (see Table 1 and Fig. 1 for details). Three of seven subjects had a right cortical AVM, three had left cortical AVM, and one had a left cerebellar AVM. The location of the AVM (right or left) was defined as the affected hemisphere, while the opposite hemisphere was defined as the unaffected hemisphere.

### MRI acquisition

All 8 subjects had sagittally-acquired 3D T1-weighted magnetization-prepared rapid gradient echo (MPRAGE) sequences and 3D T2-weighted sampling perfection with application optimized contrast using different angle evolutions (SPACE) on a Siemens 3 T TIM Trio MRI scanner. A generalized autocalibrating partially parallel acquisition (GRAPPA) factor of 2 was used for both scans with 50 % phase oversampling for the MPRAGE and no oversampling for the SPACE sequences, as previously described by Glasser and Van Essen [22].

### Segmentation and registration

The T1w images were segmented using Freesurfer version 5.1.0. The resulting surfaces were registered to the fs_LR atlas and sulcal depth maps generated using previously described methods [23]. Segmentations of the AVMs were manually traced using MRIcron [24].

### Sulcal depth analysis

The PALS-B12.LR mean sulcal depth [25] was resampled to the fs_LR mesh using caret5 with an existing PALS-to-Conte69 deformation map [22]. This was the average of the sulcal depth maps for twelve human subjects, all healthy, right-handed adults aged 18–24 (six female, six male). For each vertex within each hemisphere, the difference between the subject’s sulcal depth and the PALS-B12.LR mean sulcal depth was computed. The resulting depth difference reflects the degree to which cortical depth differs from the population mean at any given vertex. Figure 1 shows depth difference maps for each subject. For each hemisphere, a depth difference index (DDI) was computed by integrating across the surface (sum of the difference multiplied by a third of the area of the vertex’s tiles), but excluding vertices in the fs_LR medial wall, and then dividing by the surface area outside of the medial wall. These DDIs were computed for both affected and unaffected hemispheres, and then input to a paired *t*-test.

In some subjects, cortex near the AVM was abnormal enough to perturb cerebral hull generation, which in turn confounded sulcal depth computation. Inspection of all hemispheres revealed that the confounds affected cortex within 15 mm of the AVM, so we restricted our DDI computation to vertices farther than 15 mm from the AVM boundary. For the unaffected hemisphere, the same ROI was used, to exclude equivalent cortex from the measure on the unaffected hemisphere (left and right hemispheres are in register with one another in fs_LR standard mesh surfaces).

### Coordinate distance analysis

**Fig. 2:**
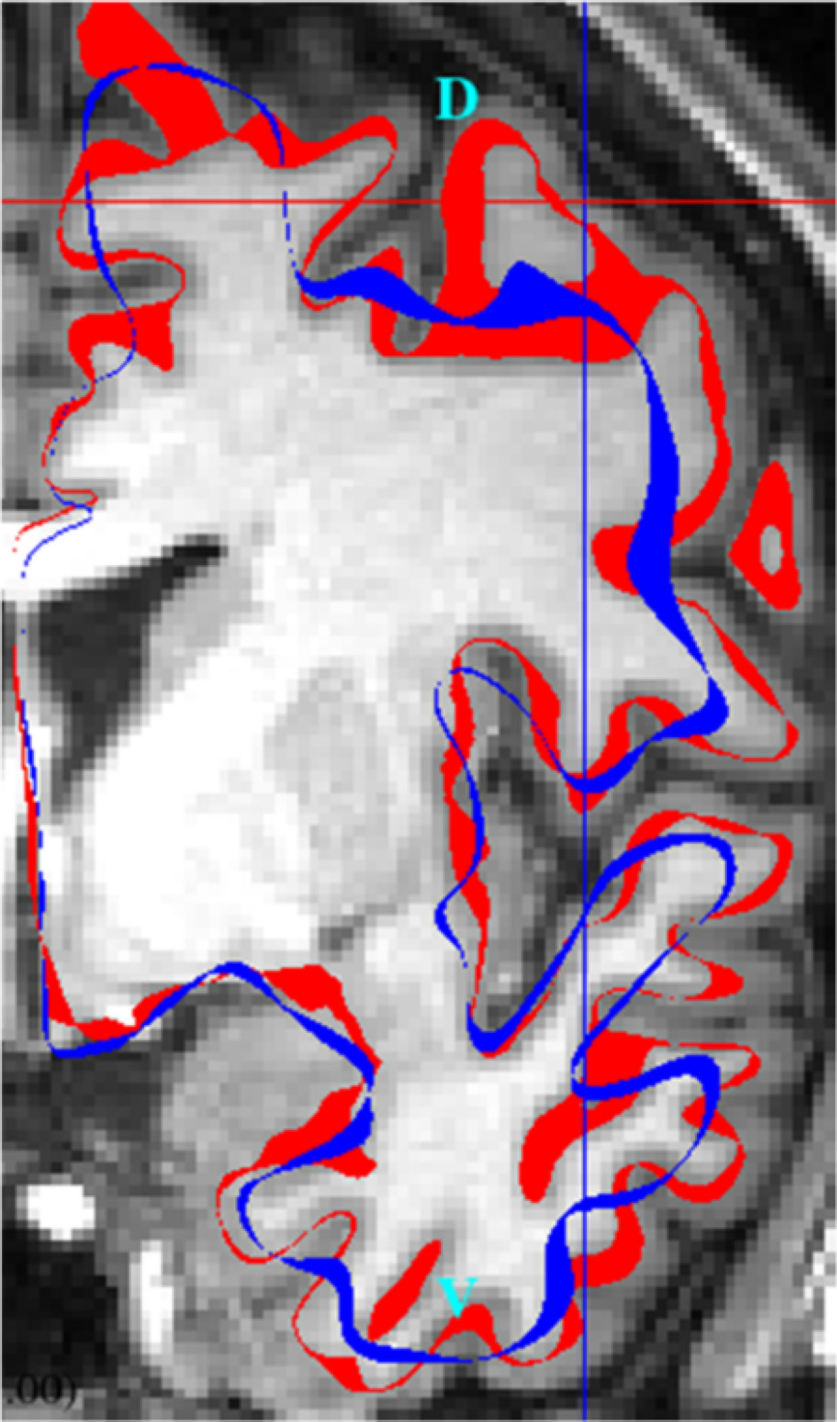
A Representation of Cortical Folding Measures. This is a coronal MRI slice of patient RD05. The red line represents the subject’s midthickness surface in Montreal Neurological Institute space. The blue line represents the HCP196 mean midthickness surface. D = dorsal; V = ventral

For this analysis, the per-vertex Euclidean distance between a normative reference average midthickness surface and the subject’s own Montreal Neurological Institute (MNI)-space midthickness surface was used as a measure of folding abnormality. Figure 2 illustrates a patient’s MNI-space surface, red, versus the normative reference midthickness, blue, in a coronal MRI slice.

The normative reference (RefSurf) was the mean midthickness surface from the Human Connectome Project (HCP) third release (Q1-2-3_Related196.L/R.midthickness.MSMSulc.164k_fs_LR.surf.gii), incorporating 196 healthy human young adults [26]. Each subject’s midthickness surface (SubjectSurface) was normalized to MNI space by applying the talairach.xfm from the subject’s freesurfer directory.

For each vertex i within each hemisphere, the distance between RefSurf[i] and SubjectSurface[i] was computed. For each hemisphere, a weighted sum of distance was computed across the hemisphere (sum of the distance multiplied by the mean of the area of the vertex’s tiles), but excluding vertices in the fs_LR medial wall. Because this measure was not subject to the hull issues that limited DDI computation to vertices beyond 15 mm from the AVM, we computed it across two regions of interest: One local (within 15 mm of the AVM) and one remote ROI (further than 15 mm from the AVM). We hypothesized that differences in the affected hemisphere might be more pronounced near the AVM. This measure, the coordinate distance index (CDI), was computed by dividing the weighted sum of distance by the surface area of the associated region. CDIs were computed for both affected and unaffected hemispheres and then input to a paired *t*-test.

### Gyrification index analysis

Gyrification index (GI) is defined as the ratio of cortical surface area to cerebral hull surface area [25, 27]. GI was computed for each subject, and then the GI for the affected and unaffected hemisphere was compared with a paired *t* test.

### Power analysis

We assume and a one-tailed test with statistical significance, α = 0.05 and a study power of β = 0.80. For a moderate effect size (50 %), we would need 21 participants (G*Power 3.1, [28]).

## Results

In order to test whether the affected hemisphere had alterations in cortical folding relative to the unaffected hemisphere, we utilized 3 metrics - depth difference index (DDI), coordinate distance index (CDI) and gyrification index (GI). This data is presented in Table 2. With our small sample size of 7 subjects, none of these three metrics had a significant difference between the affected and unaffected hemisphere.

**Fig. 3:**
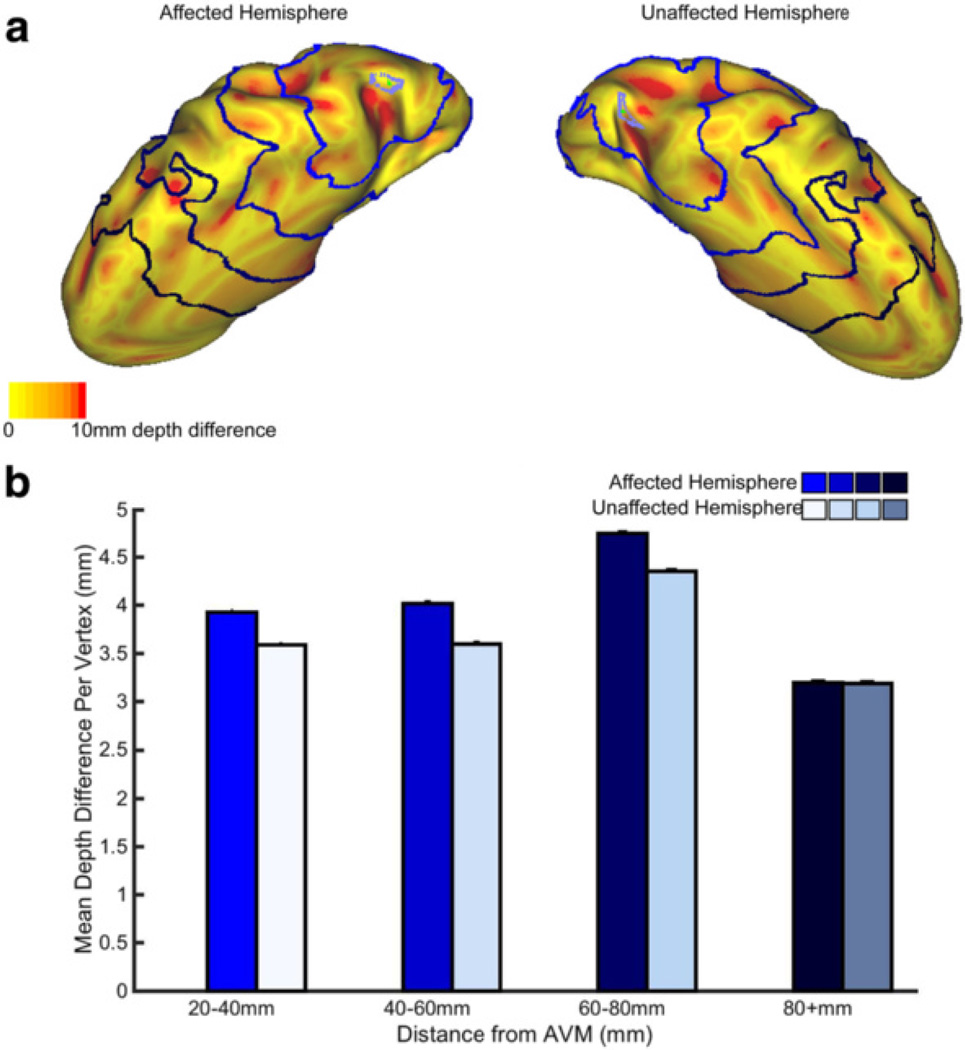
The Effect of the AVM on Sulcal Depth Difference from the Population Mean for an Example Subject. **a** Depth difference for each vertex is displayed using color intensity from yellow-red on the inflated surface for subject RD05. Surfaces were rotated to optimally view the area of the AVM and directly surrounding in; in this case, the hemispheres are visualized from the top-down (see RD05’s scan in Fig. 1 to aid with orientation). The blue rings correspond to distance from the AVM outline. For the unaffected hemisphere, rings correspond to the distance to the AVM if it were in that hemisphere (“flipped” AVM). Depth difference maps for all other subjects are in Fig. 4. **b** Mean depth difference is calculated for bands at varying distance from the AVM for subject RD05. Bar graphs represent the mean depth difference at each band: 20–40 mm, 40–60 mm, 60–80 mm and 80 + mm. Colors on the bar graph represent the band just outside of the corresponding color rings in 3a. Unaffected hemisphere values are on the right, and are shown in a lighter color to aid viewing. Plots for all other subjects are in Fig. 5. Error bars are SEM

**Fig. 4:**
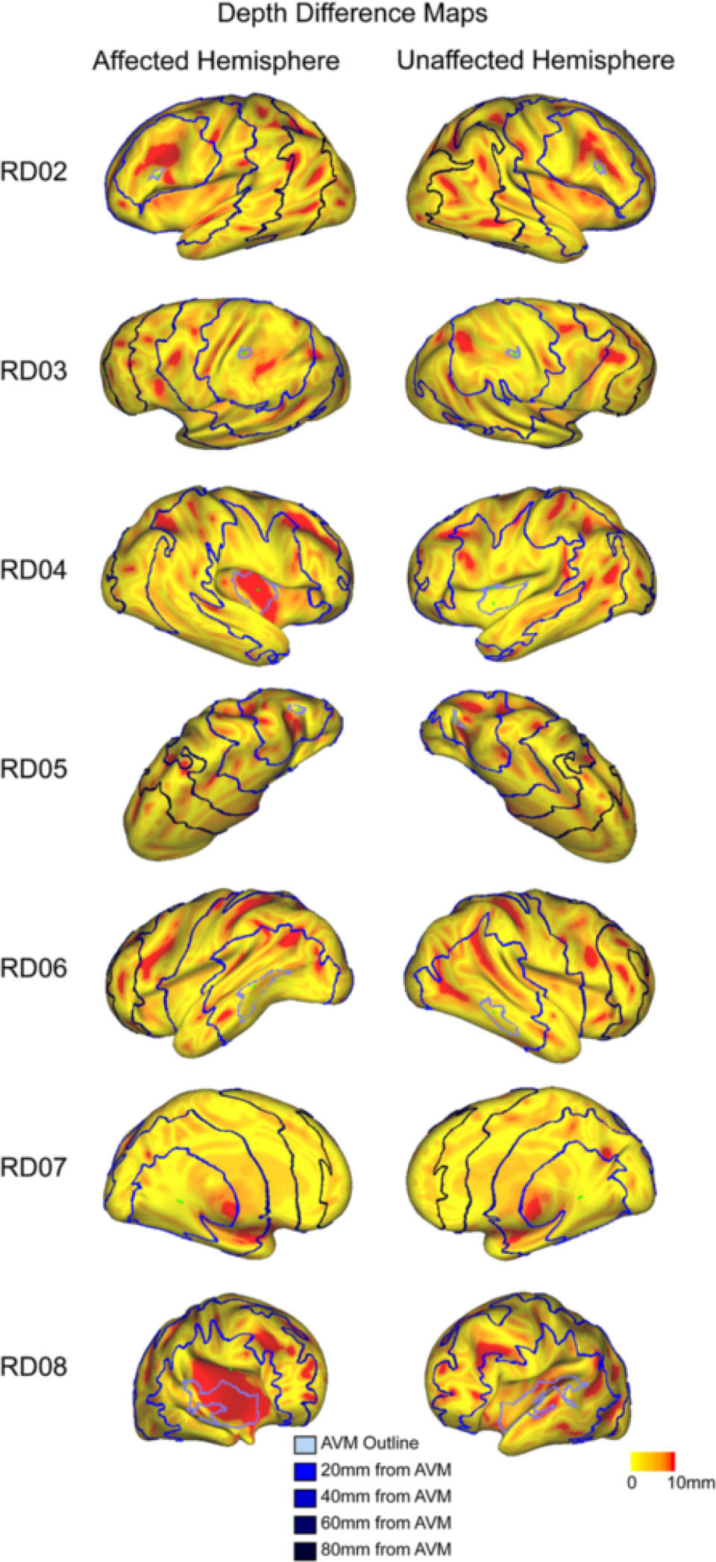
Depth Difference Maps for Every Subject. Depth difference for each vertex is displayed using color intensity from yellow-red on the inflated suface for every subject. Surfaces were rotated to optimally view the area of the AVM and directly surrounding in (see scan in Fig. 1 to aid with orientation). The blue rings correspond to distance from the AVM outline. For the unaffected hemisphere, rings correspond to the distance to the AVM if it were in that hemisphere (“flipped” AVM)

**Fig. 5:**
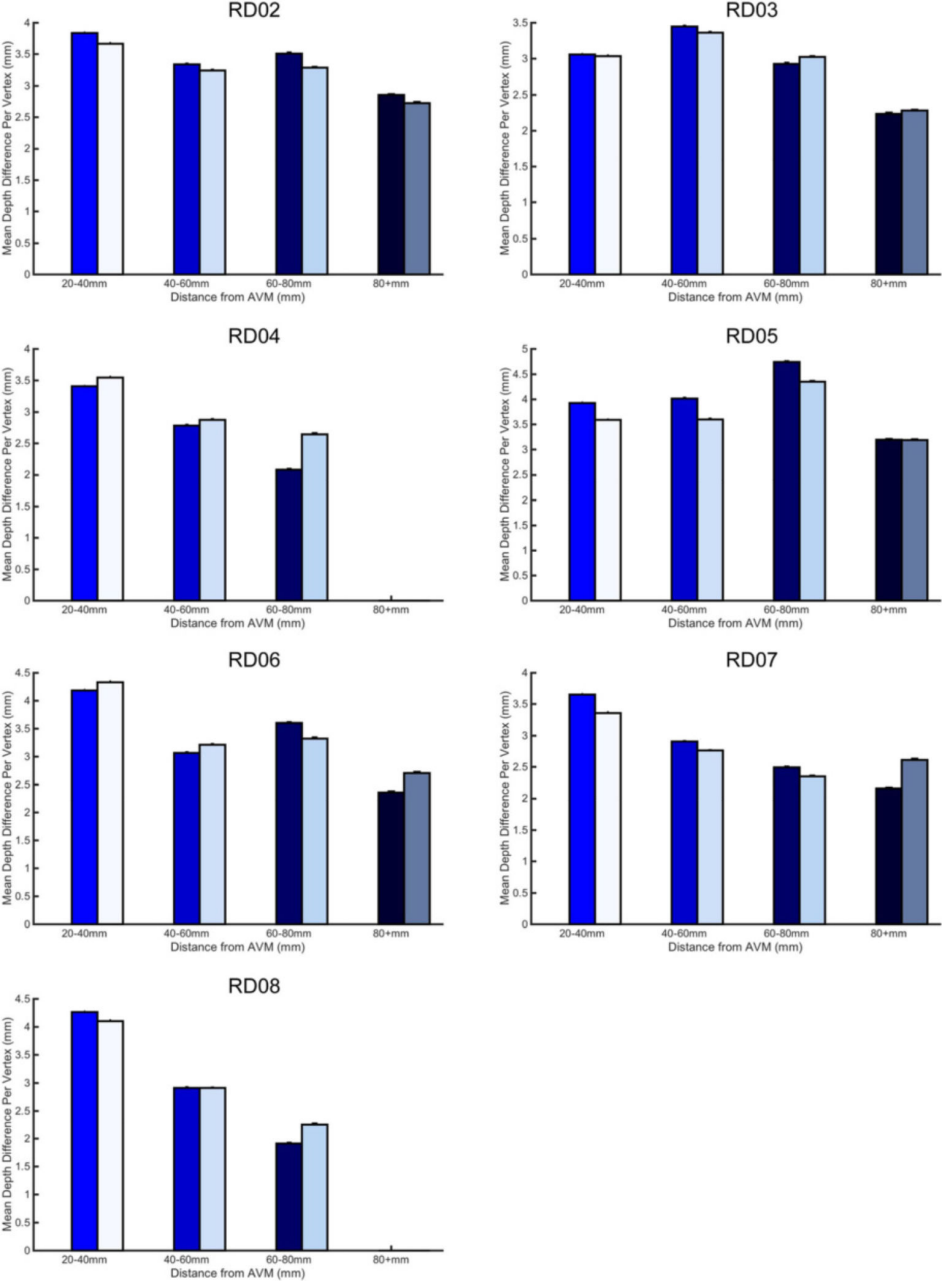
Relationship between Distance from the AVM and Depth Difference for every subject. Mean depth difference is calculated for bands at varying distance from the AVM. Bar graphs represent the mean depth difference at each band: 20–40 mm, 40–60 mm, 60–80 mm and 80 + mm. Colors on the bar graph represent the band just outside of the corresponding color rings in Fig. 4. Unaffected hemisphere values are on the right, and are shown in a lighter color to aid viewing. Error bars are SEM

We next examined the relationship between depth difference (DDI prior to integration across all vertices) and distance from the AVM center. An example subject depth difference map is shown in Fig. 3a (maps for each subject are shown in Fig. 4). Qualitatively, variability makes it difficult to discern a spatial pattern for depth difference. We drew regions of interest (ROIs) in 20 mm bands starting 20–40 mm from the AVM (ROIs illustrated in Fig. 3a). We observed that most subjects had a difference between affected and unaffected sulcal depth which was more pronounced in ROIs closer to the AVM (see example in Fig. 3, RD05). Nonetheless, the existence of subjects for which this trend does not hold (ex. RD06, Fig. 5) indicates that other variables, such as AVM depth and location, may also affect cortical folding.

**Fig. 6:**
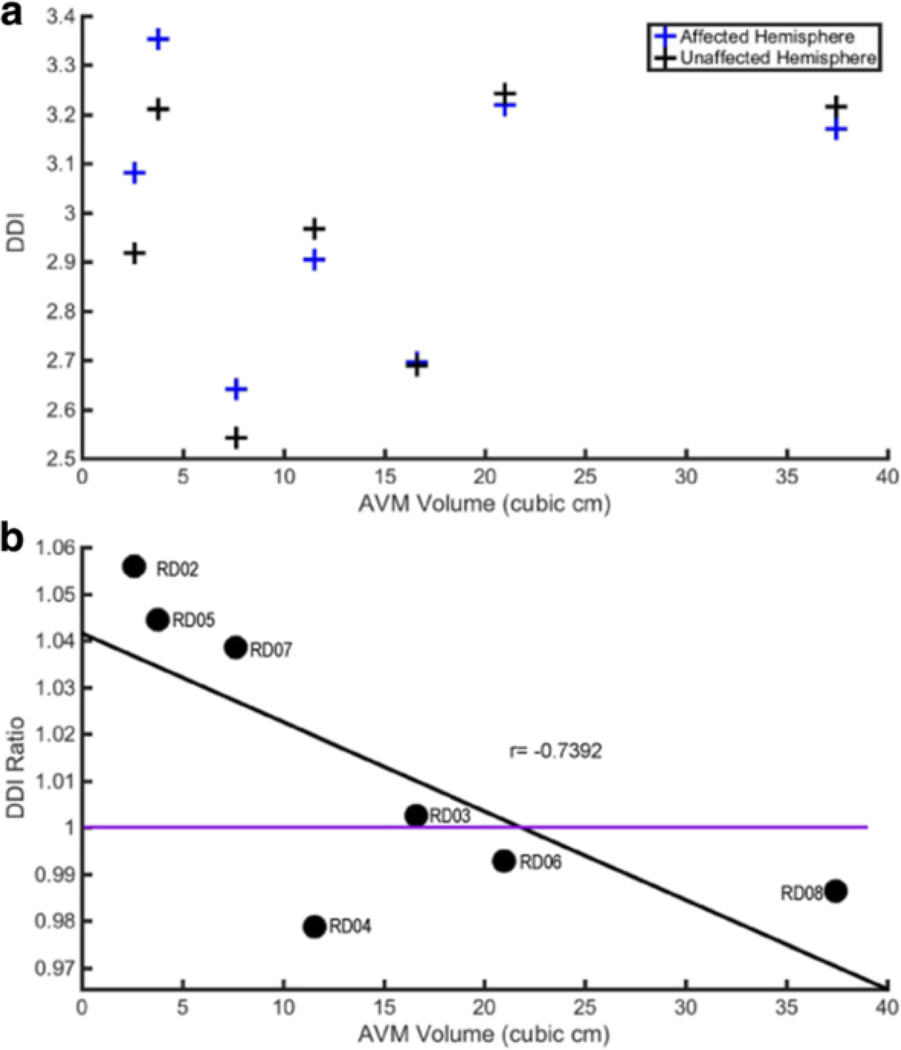
The Relationship of AVM Volume with Integrated Depth Difference (DDI). **a** DDI (mm) plotted against AVM volume (cubic cm) for each subject. The affected hemisphere is represented in blue, unaffected in black. **b** DDI Ratio is plotted against AVM volume (cubic cm). DDI ratio is the ratio of affected/unaffected DDI (i.e. the 2 points for each subject in **a**). The subject identifier is shown next to each data point. A linear regression line is shown in black to demonstrate the negative correlation (*r* = −0.74), and the purple line is DDI Ratio = 1 (affected = unaffected)

We also looked at whether there was a relationship between AVM volume and DDI. Fig. 6a is a scatter plot of AVM volume and DDI for the affected and unaffected hemispheres from each subject. There is a tight correlation in normalized sulcal depth between left and right hemispheres within a subject, which seemed to mask any effects of the AVM volume. To address this, we calculated the ratio of affected:unaffected DDI and compared this to AVM volume (Fig. 6b). Interestingly, with just 7 data points, this DDI ratio was inversely correlated with AVM volume (correlation coefficient *r* = −0.74, *p* = 0.04).

## Discussion

This is the first morphometric study to quantitatively assess brain cortical folding differences in human subjects with brain arteriovenous malformations using Human Connectome Project (HCP) methodology [11, 22, 23]. We quantitatively assessed brain sulcal depth in 7 participants with AVMs compared to the mean depth of healthy young adults. We found no statistically significant differences between hemispheres containing the AVM and contralateral hemispheres in our sulcal depth analysis, coordinate distance analysis or gyrification index testing. However, the depth difference index (DDI) ratio (affected hemisphere/unaffected hemisphere) was larger in smaller AVMs and smaller in larger AVMs (both hemispheres had similar DDI). One hypothesis for why this may be occurring is that the larger AVMs are more likely to exert effects on the contralateral as well as ipsilateral hemisphere. However, as with distance from the AVM, the occurrence of significant outliers in this trend suggests that AVM volume is not the only factor influencing sulcal depth. The complexities of the interaction between AVM pathogenesis and cortical development are deserving of further study. Currently, the only mechanistic understanding of AVM pathogenesis comes from the observation of increased intracranial AVM prevalence in the main subtypes of an autosomal dominant syndrome with AVMs in various organs including the brain: Human Hereditary Telangiectasia (HHT1 and HHT2). HHT1 involves a mutation in endoglin (Eng) and HHT2 involves a mutation in actin-like kinase 1 (Alk1), both involved in the transforming growth factor beta (TGF-**β**) signaling cascade [29, 30]. As both Eng and Alk1 are expressed in endothelial cells, changes in their function and expression affect angiogenesis [31, 32]. A murine knockout of Alk1 needed angiogenic stimulation with VEGF for *de novo* formation of brain AVMs [33], potentially suggesting a “two-hit” model of AVM formation and explaining the relatively few congenital AVM cases. The vast majority of brain AVMs are present in the absence of HHT and there are no definitive human tissue studies implicating Alk1 in brain AVM formation [34, 35], leaving a major gap in the understanding of AVM pathogenesis.

Our results may support the *de novo* formation theory, as otherwise cortical folding differences would potentially be more evident. However, with only 7 patients at a single time instance, more longitudinal data is needed for such a conclusion. We originally hypothesized that sulcal depth differences would be confined to the cortex adjacent to the AVM. The AVM should provide local mechanical traction to folding and also a relative lack of perfusion caused by the absence of normal intervening capillaries. We were unable to define a spatial relationship of cortical folding differences caused by underlying AVMs, an important result that may suggest a decoupling of cortical folding and AVM pathogenesis. This lack of spatial relationship could be due to a much wider-spread pattern of cortical disruption, but it could also be that folding and AVM formation processes are independent.

Previous studies [36] have shown reorganization in eloquent areas overlying AVMs, which could have its basis in cortical folding but also in post-stroke cortical plasticity mechanisms that are also poorly understood [37]. Additionally, it is possible that extreme sulcal depth differences predispose AVM patients to ipsilateral seizure onset during the initial presentation in nearly 40 % of AVM patients [38], as many epilepsy etiologies are structural [39]. However, every healthy individual has a distinct sulcal depth pattern and our comparisons employ the population mean. Further testing of sulcal depth and functional changes could further elucidate this relationship, especially if performed longitudinally in our AVM patient cohort.

**Fig. 7:**
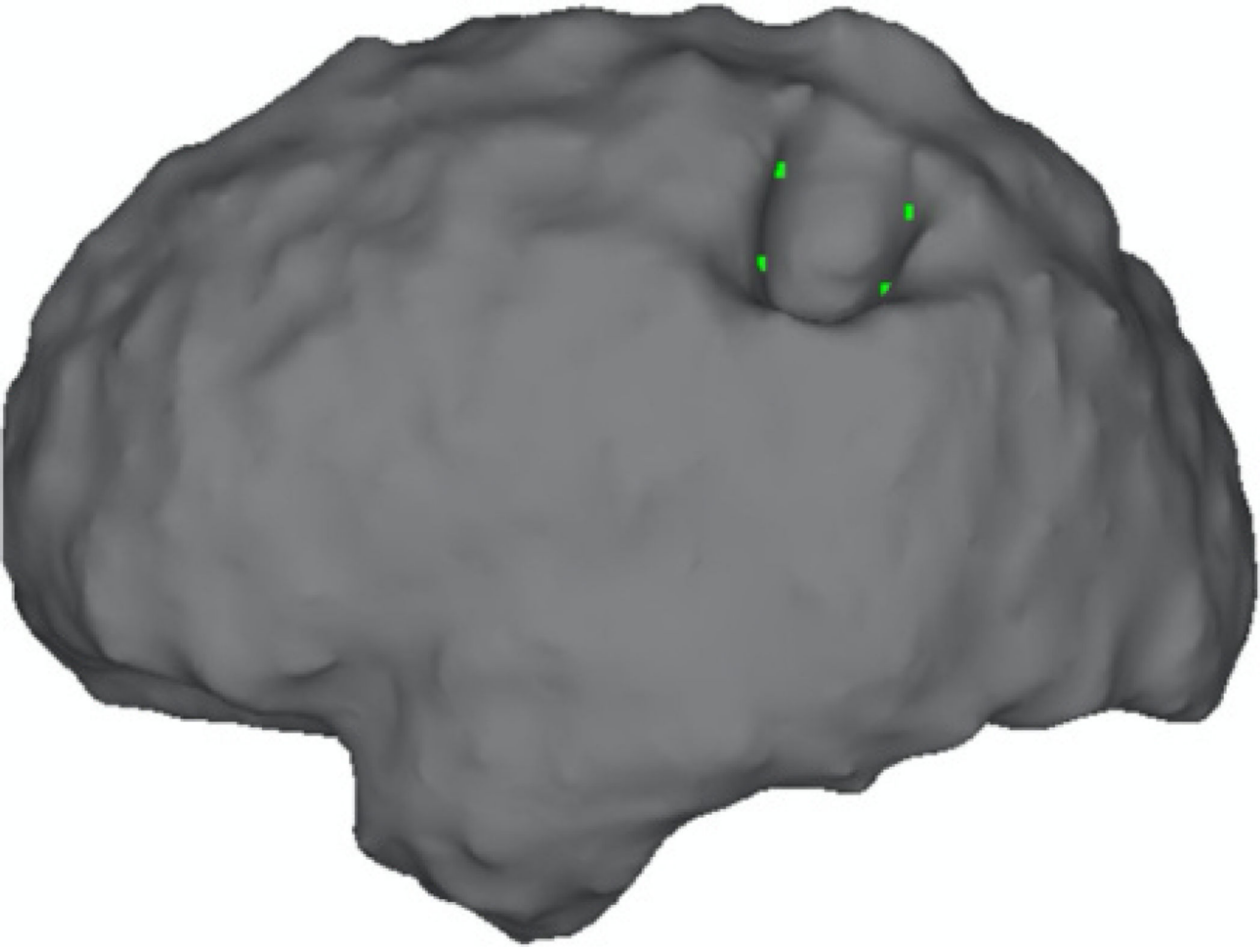
Example Cerebral Hull Defect Related to AVM. Invagination in Convex Hull for Case RD03’s Left Hemisphere

Despite a large body of scientific literature, the pathogenesis of AVMs remains controversial [40, 41]. Our study raises several points essential to AVM pathogenesis. Although classically described as congenital malformations, there now exist many case reports of *de novo* AVM formation [42] on serial cerebral angiography. Our study represents a single point in time for each patient, and any structural differences seen in our population could be a result of a disordered cerebral circulation. However, there may be artifact in our calculation of sulcal depth related to gliosis developed alongside a *de novo* AVM and the subsequent changes in perfusion to the surrounding tissues. Such remodeling has previously been described in patients with cerebrovascular disease [43]. A longitudinal study looking at the evolution of sulcal depth differences in patients with AVMs could be illuminating. The sulcal depth measure was confined to vertices outside a 15 mm radius of the AVM, due to problems generating the lesion’s convex hull for analysis; Fig. 7 demonstrates a representative AVM-related hull defect. The presence of the AVM during cerebral hull generation in our study patients also likely confounds the gyrification index calculation. Morphometric measures that depend on nonlinear registration to an atlas target (e.g., deformation-based morphometry) may have similar confounds, to the extent that the AVM affects registration. Cortical thickness might be less affected overall and could be examined in future studies.

Our study might be simply underpowered to determine such a relationship with only six subjects with supratentorial AVMs. At our hypothesized moderate 50 % effect size, we would have needed 21 patients to have 80 % power to avoid a type II error. In addition to limiting the power of our study of sulcal depth differences, none of these patients presented with hemorrhage or focal neurological deficit. It is possible that these sequelae are in some way related to the minor folding differences we observed, but a larger study would be needed to determine what, if any, connection there is between the two.

## Conclusions

This is the first study to quantitatively assess human developmental cortical folding in the presence of intracranial arteriovenous malformations. The study found no statistically significant cortical folding differences in the patient hemispheres with AVMs compared to their own contralateral hemispheres or compared to a previously obtained healthy, control dataset. However, longitudinal studies are recommended to definitively establish whether arteriovenous malformations are developmental, congenital lesions or dynamic, *de novo* entities.

## Figures and Tables

**Table 1:** Characterization of the Seven AVM Subjects. This table indicates the cohort demographics, AVM characteristics, presentation, treatment and follow-up. Yrs = years, F/U = follow-up, mo = months

Subject	Age (yrs) /Gender	Grade	Location	Volume (cm3)	Presentation	Treatment	F/U (mo)	Outcome
RD02	46/M	4	Left Frontal	2.62	Incidental	Radiation	0	N/A
RD03	40/M	3	Left Motor	16.6	Seizures	Radiation	44	Response
RD04	43/M	3	Right Insular	11.5	Numbness	Radiation	39	Response
RD05	22/F	3	Right Motor	3.77	Incidental	None	0.1	N/A
RD06	47/M	4	Left Parietal	21.0	Headaches	Radiation	5.5	Expired
RD07	56/F	2	Vermian	7.63	Incidental	Surgery	18	Response
RD08	41/F	4	Right Temporal	37.4	Incidental	None	19	Growth

**Table 2:** Depth difference index (DDI), local (L) and remote (R) coordinate distance index (CDI and CDI), and gyrification index (GI) for Affected (A) and Unaffected (U) Hemispheres. Both DDI and CDI are vertex-wise differences (affected-unaffected) weighted by mean tile area and summed over vertices outside the medial wall (for DDI, vertices at least 20 mm from AVM)

Subject	DDI (A)	DDI (U)	L CDI (A)	L CDI (U)	R CDI (A)	R CDI (U)	GI (A)	GI (U)
RD02	3.081085	2.917788	5.818325	7.726299	6.756877	6.732552	2.1584	2.1512
RD03	2.696007	2.689072	7.650329	6.760775	6.052032	6.439287	2.1486	2.1609
RD04	2.905195	2.967963	8.866142	5.810119	9.404687	7.920285	1.9756	1.8767
RD05	3.352648	3.210038	7.815037	9.577932	7.498025	8.160805	2.2231	2.1589
RD06	3.219622	3.242632	12.84460	7.324401	11.34821	10.15757	1.5190	1.5667
RD07	2.640733	2.542771	5.383245	5.923716	5.747210	6.088752	1.8974	1.9363
RD08	3.171523	3.215196	11.91789	9.246596	13.68615	13.38209	1.5644	1.7112
Mean	3.009545	2.969351	8.613653	7.481405	8.641884	8.411620	1.9266	1.9374
